# Progress in Immunization Information Systems — United States, 2011

**Published:** 2013-01-25

**Authors:** Cristina Cardemil, Laura Pabst, Ken Gerlach

**Affiliations:** Immunization Services Div, National Center for Immunization and Respiratory Diseases, CDC

Immunization information systems (IIS) are confidential, computerized, population-based systems that collect and consolidate vaccination data from vaccination providers and provide important tools for designing and sustaining effective immunization strategies (1,2). A *Healthy People 2020* objective (IID-18) is to increase to 95% the proportion of children aged <6 years whose immunization records are in fully operational, population-based IIS (3). The National Vaccine Advisory Committee (NVAC) has published goals for IIS, including required and optional core data elements for which IIS should collect information (4,5). Two of the required core data elements are vaccine manufacturer and vaccine lot number. To monitor progress toward achieving these and other program goals, CDC annually surveys 56 immunization program grantees using the IIS Annual Report (IISAR). Results from the 2011 IISAR (completed by 54 grantees) indicate that 84% (19.2 million) of U.S. children aged <6 years participated in IIS, as defined by having at least two recorded vaccinations, an increase from 82% (18.8 million) in 2010 (1). Grantees reported that an average of 63% of vaccination records for these children contained data in the field for vaccine manufacturer and 60% contained data in the field for lot number. A new project under way to capture vaccine product information, expiration date, and lot number on two-dimensional (2D) barcodes on vaccine vials might increase completeness, accuracy, and availability of these data elements in patient medical records and IIS, which in turn might enhance vaccine safety and support vaccine inventory management.

Of the 56 immunization program grantees (50 states, five cities,* and the District of Columbia), 2011 IISAR data† were available for 54 grantees. Connecticut did not report, and New Hampshire was not eligible because it did not have an IIS in 2011. The self-administered survey asked about participation in IIS, data quality indicators, and IIS functionality (e.g., interoperability with electronic health records, vaccine inventory management, and use of IIS data).

## Child Participation in IIS

Child participation was defined as having two or more vaccinations documented in an IIS. Participation was calculated by dividing the number of children aged <6 years in an IIS who met this criteria by the 2011 U.S. Census estimate for the number of children of the same age group in the grantee’s geographic area (6). Demographic data in IIS initially are obtained from birth certificates and birth hospital records, which often also contain records of the birth dose of hepatitis B vaccine. Defining participation in IIS as having ≥2 recorded vaccine doses makes it highly likely that the child received at least one vaccination from a provider other than the birth hospital who participates in IIS.

**FIGURE 1:**
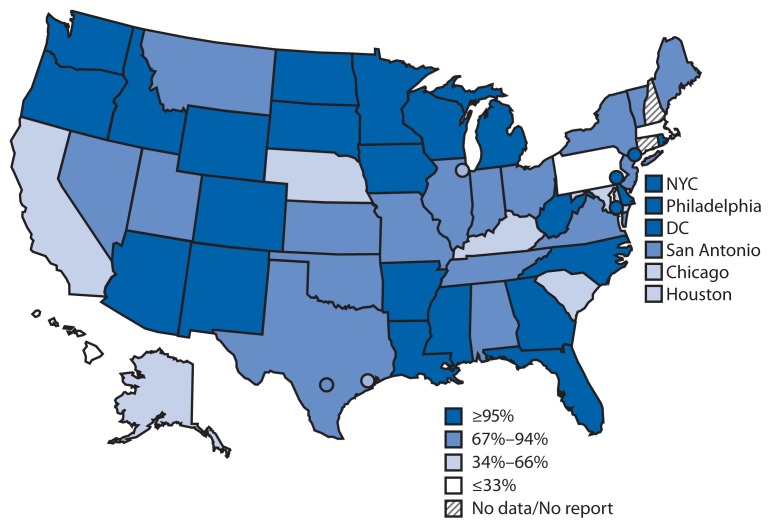
Percentage of children aged <6 years participating* in an Immunization Information System — United States, five cities,^†^ and the District of Columbia, 2011 Abbreviations: NYC = New York City; DC = District of Columbia. * Defined as having two or more vaccinations recorded in the IIS. ^†^ Chicago, Illinois; Houston, Texas; New York, New York; Philadelphia, Pennsylvania; and San Antonio, Texas.

Nationally, 19.2 million U.S. children aged <6 years (84%) participated in an IIS in 2011. Child participation in IIS has increased steadily, from 63% in 2006 (1) to 84% in 2011. Of the 54 grantees with available data in 2011, 24 (44%) reported that >95% of children aged <6 years in their geographic area participated in their IIS. An additional 13 (24%) grantees reported child participation rates ranging from 80% to 94% (Figure 1).

## Core Data Elements

Each year, CDC collects IISAR information on the percentage of grantees meeting 12 NVAC functional standards for IIS (4,5). The initial standards were developed in 2001; NVAC revised and published new IIS functional standards in 2007. Progress in meeting functional standard 1, electronically store data on NVAC-required core data elements,§ is reported here. For each of the 12 NVAC-required core data elements, grantees report if their IIS contains a field for that element and, for those that do, the percentage of records¶ belonging to children aged <6 years that contain data in the field. Three of the 12 core data elements contain multiple components for a total of 18 data elements. These data are used to calculate an unweighted U.S. national average for field completeness.

**FIGURE 2:**
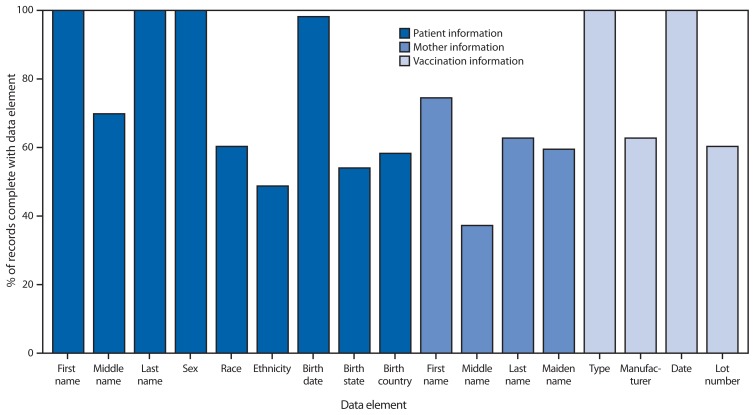
Percentage of vaccination records for children aged <6 years complete with required National Vaccine Advisory Committee core data elements — United States, 2011

Among the 54 grantees reporting in 2011, 32 (59%) included a field for each of the 18 data elements in their IIS. The most common data elements not included in IIS were birth order for multiple births only (nine grantees), mother’s middle name (eight grantees), and birth country (six grantees). Average completeness of NVAC core data elements for children aged <6 years ranged from 38% for mother’s middle name to ≥98% for six fields (patient’s first name, last name, sex, birth date, vaccine type, and vaccination date) (Figure 2).

**FIGURE 3:**
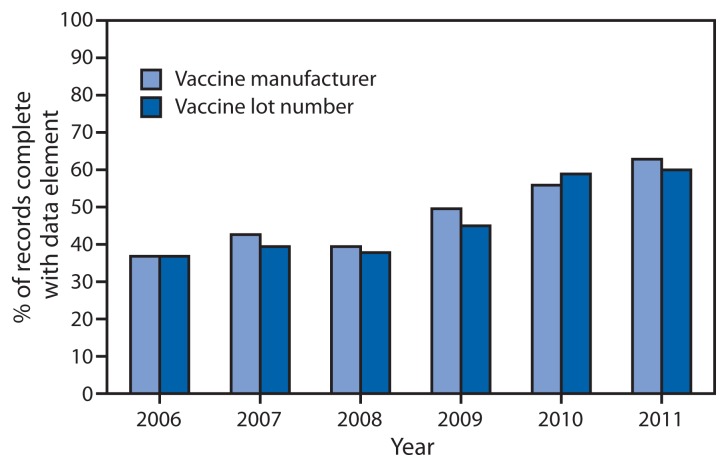
Percentage of vaccination records for children aged <6 years containing vaccine manufacturer and lot number in immunization information systems — United States, 2006–2011

Vaccine manufacturer and vaccine lot number are two data elements recognized for their importance in IIS for vaccine inventory management and their potential to increase patient safety through identification of persons who were administered recalled vaccine and reporting of vaccine-associated adverse events. The proportion of IIS including fields for these data elements in IIS has been high. In 2006, 89% of IIS contained a field for recording vaccine manufacturer and 88% contained a field for lot number; these increased in 2011 to 98% and 100%, respectively. The completeness of data in these fields has increased from 37% for both in 2006 to 63% for vaccine manufacturer and 60% for lot number in 2011 (Figure 3).

### Editorial Note

Child participation in IIS and completeness of data for vaccine manufacturer and lot number in IIS increased steadily from 2006 to 2011. Despite this progress, challenges remain to meeting the *Healthy People 2020* objective for child participation to increase to 95% the proportion of children aged <6 years whose immunization records are in fully operational, population-based IIS, and completeness of the vaccine fields remains suboptimal. Maximal child participation and complete records are needed to fully realize the benefits of IIS, which include clinical decision support, vaccination coverage reports, support for vaccine-preventable disease outbreak response, vaccine inventory management, and the ability to generate reminder and recall messages. Developing and promoting these beneficial tools that IIS offer to providers can encourage provider participation in IIS. If provider participation in IIS increases, child participation increases.

Several ongoing initiatives are expected to increase participation in IIS and improve the completeness and accuracy of data contained in IIS, including enhanced interoperability between IIS and electronic health records (EHRs), the use of IIS to interface with VTrckS, CDC’s vaccine tracking system for publicly purchased vaccine, and the use of 2D barcodes to facilitate recording vaccine information in EHRs and IIS. VTrcks and interoperability initiatives have been reported on previously (1,7). The 2D barcode project is an additional initiative that might help to address the completeness and accuracy of these fields.

In September 2011, CDC initiated a 2D vaccine barcode pilot project** to assess the impact and best practices of 2D barcoded vaccines on vaccine administration and inventory management. This pilot project also will assess the ability of 2D barcoding technology to improve the completeness and accuracy of electronically stored immunization information. Printed 2D barcodes encode more information in a smaller area than the space needed for linear barcodes, meaning that all vaccine product data can be encoded into a symbol compact enough to appear on a single-dose vial or syringe. Although the linear barcode could hold the information, space constraints on the vial or syringe make the use of the linear barcode unrealistic. A 2D barcode will contain a Global Trade Identification Number that uniquely identifies the product and manufacturer, the lot expiration date, and the lot number. Pilot participants include 10 CDC immunization program grantees, 220 immunization providers (public and private), and two vaccine manufacturers.

What is already known on this topic?

In 2010, 82% of the 18.8 million U.S. children aged <6 years participated in immunization information systems (IIS).

What is added by this report?

In 2011, 84% (19.2 million) of U.S. children aged <6 years participated in IIS. Among IIS grantees, completeness of data for vaccine manufacturer and vaccine lot number has increased since 2006, but remained suboptimal at 63% and 60%, respectively, in 2011. In 2011, CDC initiated a vaccine barcoding pilot project to determine best practices for labeling and tracking vaccines using 2D barcodes that include vaccine product information, lot number, and expiration date.

What are the implications for public health practice?

Widespread adoption of 2D vaccine barcoding among manufacturers and providers has the potential to increase completeness and accuracy of IIS data and improve patient safety. More complete and accurate data elements might provide an additional incentive for providers to participate in IIS, which in turn can increase child participation in IIS.

Documenting vaccine product information and lot number is required by the National Childhood Vaccine Injury Act of 1986, and the American Academy of Pediatrics recommends documenting vaccine expiration date to improve patient safety. To report vaccine product data to an IIS, providers must either type them into an EHR, which transmits data to the IIS, or type the information into the IIS directly. In addition to recording data electronically in an EHR or IIS, some providers also record these data by hand in paper records. Recording information by hand and duplication of entries takes time and resources and increases the likelihood of data entry errors.

Use of a 2D barcode on vaccines could allow for rapid, complete, and accurate capture of these vaccine product data by a barcode scanner that could transfer the information to EHRs and IIS. A CDC assessment of the impact of 2D barcode for vaccine production, clinical documentation, and public health reporting and tracking analyzed the expected costs and benefits of barcode use by immunization providers. The assessment found that for every $1 expended, $2.70–$2.80 in benefits were expected to accrue from 2011 through 2023 (8). Net benefits to society were forecast to be $326 million to $349 million. Among surveyed primary-care providers who did not report immunizations to IIS currently, 63% indicated they would be more likely to do so if the 2D barcode were available.

The findings in this report are subject to at least two limitations. First, data from the IISAR were self-reported and self-validated. Second, because two of the 56 grantees did not report data during the period of data collection, the nationwide IIS participation rates for children aged <6 years and completeness of core data elements might be underestimated or overestimated.

As with other technological advances, adoption of 2D barcode technology for recording vaccination information has the long-term potential to improve vaccine safety monitoring and inventory management, reduce staff time spent manually capturing vaccine data, decrease costs for vaccine stakeholders, and enhance the completeness and accuracy of vaccination information in electronic medical records and IIS. The barcode effort is one of many recent advances in health-care technology that should have the added benefit of reducing the reporting burden on providers while improving the quality of data in IIS. These advances and the resulting increase in data quality might lead to increased vaccine provider, and therefore child, participation in IIS, and achievement of the *Healthy People 2020* objective and NVAC goals.

## Footnotes

* Chicago, Illinois; Houston, Texas; New York, New York; Philadelphia, Pennsylvania; and San Antonio, Texas.

† Additional information available at http://www.cdc.gov/vaccines/programs/iis/annual-report-iisar/index.html.

§ Required core data elements include the patient’s name (first, middle, last), sex, race, ethnicity, birth date, birth state/country, birth order, mother’s name (first, middle, last, maiden), vaccine type, vaccine manufacturer, vaccination date, vaccine lot number. Data on birth order is not reported here because field completeness can only be assessed if a person is part of a multiple birth, which is a relatively infrequent event.

¶ IIS contain one record per child and a field for each data element pertaining to a patient’s and mother’s demographic information. Each child has multiple vaccination records, and each of these vaccination records has an associated field for each data element pertaining to that vaccination (e.g., manufacturer, date, and lot number).

** Additional information available at http://www.cdc.gov/vaccines/programs/iis/activities/2d-vaccine-barcoding.html and http://www.2dbarcodepilot.com.
